# Preoperative risk stratification models fail to predict hospital cost of cardiac surgery patients

**DOI:** 10.1186/1749-8090-8-126

**Published:** 2013-05-09

**Authors:** Akmal MA Badreldin, Fabian Doerr, Axel Kroener, Thorsten Wahlers, Khosro Hekmat

**Affiliations:** 1 Department of Aneasthesia and Operative Intensive Care Medicine, University of Bonn, Sigmund-Freud-Street 25, Bonn 53127, Germany; 2 School of Medicine, Friedrich-Schiller-University of Jena, Bachstraße 18, Jena 07743, Germany; 3 Department of Cardiothoracic Surgery, University of Cologne, Kerpenerstraße 62, Cologne 50937, Germany

**Keywords:** Hospital cost, Cardiac surgery, Scoring models

## Abstract

**Background:**

Preoperative risk stratification models have previously been suggested to predict cardiac surgery unit costs. However, there is a lack of consistency in their reliability in this field. In this study we aim to test the correlation between the values of six commonly known preoperative scoring systems and evaluate their reliability at predicting unit costs of cardiac surgery patients.

**Methods:**

Over a period of 14 months all consecutive adult patients undergoing cardiac surgery on cardiopulmonary bypass were prospectively classified using six preoperative scoring models (EuroSCORE, Parsonnet, Ontario, French, Pons and CABDEAL). Transplantation patients were the only patients we excluded. Total hospital costs for each patient were calculated independently on a daily basis using the bottom up method. The full unit costs were calculated including preoperative diagnostic tests, operating room cost, disposable materials, drugs, blood components as well as costs for personnel and fixed hospital costs. The correlation between hospital cost and the six models was determined by linear regression analysis. Both Spearman’s and Pearson’s correlation coefficients were calculated from the regression lines. An analysis of residuals was performed to determine the quality of the regression.

**Results:**

A total of 887 patients were operated on for CABG (n = 608), valve (n = 142), CABG plus valve (n = 100), thoracic aorta (n = 33) and ventricular assist devices (n = 4). Mean age of the patients was 68.3±9.9 years, 27.6% were female. 30-day mortality rate was 4.1%. Correlation between the six models and hospital cost was weak (Pearson’s: r < 0.30; Spearman’s: r < 0.40).

**Conclusion:**

The risk stratification models in this study are not reliable at predicting total costs of cardiac surgical patients. We therefore do not recommend their use for this purpose.

## Background

Preoperative risk stratification scoring models have been used to predict mortality after heart surgery [1]. Several studies have suggested that these models can be used to predict the total cost of cardiac surgical patients [2-7]. An admission scoring model that can accurately predict an estimate of patients’ cost would facilitate allocation of resources as well as the planning of more economical patient care. As these models were originally designated for the sole purpose of predicting mortality we find their use to predict events other than mortality (e.g. morbidity or hospital cost) questionable. Hence, we reviewed the literature searching for previous studies that investigated the correlation between preoperative cardiac surgical scoring models and total hospital costs. We then conducted a prospective study to evaluate these scoring systems as a predictor of total hospital costs in open heart surgery. The six models we evaluated were: EuroSCORE, Parsonnet, Ontario, French, Pons and CABDEAL.

## Methods

Risk factors for all consecutive adult patients undergoing open heart surgery in the department of Cardiothoracic Surgery at the University of Cologne, over a period of 14 months, were prospectively collected. The patients’ records contained all variables necessary to match the criteria for all six risk stratification scoring systems: EuroSCORE [8], Parsonnet [9], Ontario [10], French [11], Pons [12], and CABDEAL [13]. We have used the additive EuroSCORE and not the logistic EuroSCORE, to keep it consistent with the other models.

All information required was collected on patient admission and were documented in the quality control system QIMS 2.0b (University hospital of Muenster, Germany). Relevant laboratory data were collected electronically from the central laboratories and were integrated in the QIMS 2.0b system. The attending physicians collected the data and checked for accuracy. The data collection and calculations of the scoring systems were validated by both a medical clerk and a senior supervisor.

Total hospital costs were calculated on a daily basis for each patient using the bottom up method. All costs were calculated including preoperative diagnostic tests, operating room costs, disposables, drugs, blood components as well as costs for personnel, and hospital-fixed costs, according to the “Manual for calculating patient costs; Version 3.0” published in the year 2007 by the German Hospital Federation (DKG) and the German insurance companies (GKV and PKV).

No experimental research was conducted for this study neither on humans nor on animals. All data were measured within the limits of a routine ICU admission of each individual patient. Therefore an ethical approval was not necessary.

### Statistical methods

Statistical analyses were performed using SPSS software version 18 (SPSS Inc, Chicago, IL, USA). Continuous scale data are presented as mean ± standard deviation (SD) and were analyzed by the Kolmogorov–Smirnov test for normal distribution. A *p*-value < 0.05 was considered significant for all tests.

Linear regression analysis was used to determine the correlation between costs and the six risk stratifications models. Both Pearson’s and Spearman’s correlation coefficients were calculated from the regression line. An analysis of residuals was performed to determine the quality of the regression.

#### Pearson’s correlation

The Pearson’s correlation coefficient (r) measures the linear association between two scale variables (in this study, the score points and the total hospital cost). A perfect positive (increasing) linear correlation is represented by +1 and a perfect negative decreasing linear relationship is represented by −1 [14]. As values approach zero there is less correlation and zero represents no correlation. Generally, correlations above 0.80 are considered high.

Furthermore, the t-test is used to establish if the correlation coefficient is significantly different from zero, and, hence that there is evidence of an association between the two variables. This means that, when the *p*-value of Pearson’s test is significant, this indicates only that there is merely a linear relation between both variables. But this significance does not indicate if this relation (correlation) is high or low. This *p*-value is influenced by the sample size; however, with larger sample sizes the strength of the correlation weakens.

The normal distribution of the data in both variables is a prerequisite for Pearson’s test. If this is not the case, the conclusions may well be invalidated. Accordingly, it is better to use Spearman’s coefficient.

#### Spearman’s correlation

Rank correlation coefficients, such as Spearman’s measures the extent to which, as one variable increases, the other variable tends to increase or decreases, without requiring that this association is a linear relationship. Spearman’s test is an alternative test to Pearson’s to make the coefficient less sensitive to non-normality in distributions. However, Spearman’s test is best seen as a measure of a different type of association, rather than as alternative measure [15,16].

Unlike Pearson’s correlation, Spearman’s correlation indicates that as the value of one variable (score model in this study) changes, there is always a change in the value of the other variable (total hospital cost), when there is a perfect rank correlation. On the other hand, Pearson’s correlation is perfect when the increase in the first variable is accompanied with an equal change (increase or decrease) in the other variable. Accordingly, a good Spearman’s correlation does not necessarily mean a good Pearson’s correlation as well. Hence, the values of the two coefficients cannot meaningfully be compared [15].

## Results

A total of 887 patients were operated on with cardiopulmonary bypass. Preoperative characteristics are listed in Table 1. Mean age of the patients was 68.3 ± 9.9 years, 27.6% were female. Table 2 shows the mean values of preoperative scoring models upon admission. For the whole study population, length of stay in intensive care unit (ICU) was 88.5 ± 148.8 hours (median was 49 hours range was 14–1776 hours), hospital-stay was 16.7 ± 8.9 days and the 30-day mortality rate was 4.1%. Table 3 demonstrates the ICU-stay, hospital-stay and 30 day mortality for each operative procedure separately.

**Table 1:** Preoperative patient characteristics of the study population

**Patients data**	**All patients (n = 887)**
Age (years)	68.28 ±9.85
Female	27.6%
Weight (kg)	80.55 ± 13.33
Height (cm)	171.47 ±8.48
Hypertension	82,60%
Ejection Fraction (<50%)	8.0%
Permanent pace maker	1.60%
Heart failure	0.45%
Pulmonary Hypertension	5.40%
Active endocarditis	1.20%
Unstable angina	38.90%
Aortic dissection	1.10%
Recent myocardial infarction (< 90 days)	26.2%
Preoperative cardiac massage	2.0%
Extracardiac arteriopathy	7.80%
Ventricular tachycardia or fibrillation	2.0%
Chronic Obstructive Pulmonary Disease	10.4%
Preoperative ventilation	1.6%
Creatinine > 1.6 mmol/l	10.30%
Acute renal failure	2.80%
Focal neurological deficit	11.3%
Diabetes mellitus	27.50%
Sepsis	0.20%
Previous cardiac surgery	3.2%

**Table 2:** Mean values of the preoperative risk stratification models

**Score**	**Mean**	**Standard deviation**	**Minimum**	**Maximum**
**Additive EuroSCORE**	7.12	3.19	0	20
**Cabdeal**	2.65	1.77	0	9
**French**	6.07	3.65	0	26
**Parsonnet**	13.64	8.03	0	42
**Ontario**	5.22	2.57	0	14
**Pons**	9.73	9.05	0	42

**Table 3:** Data of different operative procedures performed during the study period

**Procedure**	**n (%)**	**ICU-stay (hours)**	**Hospital-stay (days)**	**30-day mortality (%)**
CABG	608 (68.55)	71.3 ± 124.3	15.5 ± 7.9	3.10
Valve surgery	142 (16.01)	83.2 ± 92.0	15.8 ± 6.7	3.50
CABG + Valve	100 (11.27)	134.8 ± 198.4	18.6 ± 15.4	9.0
Aortic surgery	33 (3.72)	255.7 ± 320.9	19.97 ± 10.4	9.10
VAD	4 (0.45)	359.0 ± 280.0	22.75 ± 11.7	0.0
Total	887 (100)	88.5 ± 148.8	16.7 ± 8.9	4.1

Table 4 shows the total hospital costs and the reimbursement for the different operative procedures. The reimbursement covered and exceeded the total costs in all procedures except in the VAD group, in which the reimbursement covered less than 60% of the cost.

**Table 4:** Values of total hospital cost and reimbursement in Euro for different operative procedures

**Procedure**	**Total cost**	**Costs per patient**	**Total reimbursement**	**Reimbursement per patient**
CABG	6,542,864.32	10,761.29 ± 6,319.15	7,873,216.96	12,949.37 ± 6,922.07
Valve	1,992,313.96	14,030.38 ± 5,211.57	2,426,718.94	17,089.57 ± 6,635.17
CABG + Valve	1,744,030.0	17,440.30 ± 11,261.86	1,945,560.0	19,455.60 ± 9,502.96
Aortic surgery	789,729.27	23,931.19 ± 16,721.23	846,656.58	25,656.26 ± 20,727.58
VAD	330,178.6	82,544.65 ± 15,885.09	187,413.50	46,853.25 ± 37,305.62
Total	11,399,111.97	12,851.31 ± 9,428.97	13,304,405.71	14,999.33 ± 9,292.13

The correlation of all preoperative scoring models with the total hospital cost was weak with correlation coefficients ranging between 0.122 and 0.264, according to Pearson’s test and between 0.122 and 0.372, according to Spearman’s test. This is despite a significant difference from zero in both tests with a *p*-value < 0.01 all through in both tests, except for the correlation of CABDEAL score with the reimbursement, which was not different from zero (uncorrelated) with *p* = 0.05.

## Discussion

### Statement of key findings

The purpose of this study was to evaluate whether the six preoperative risk stratification models can predict total hospital costs in cardiac surgical patients. The results show that all of these models have low correlation with total hospital cost and reimbursement (r < 0.4).

### Results of previous studies

Several studies concluded that preoperative risk stratification models, initially designed to predict mortality, are also useful for predicting resource utilization in cardiac surgical patients [2-7]. Haehnel et al. [2] suggested that the Cleveland score predicts costs in cardiac surgery. Unfortunately this paper shows this finding graphically only, without giving a correlation coefficient or a *p*-value. Kurki et al. [3] also evaluated the relationship between the Cleveland score and hospital costs. Again, no information about the correlation value was provided.

**Figure 1:**
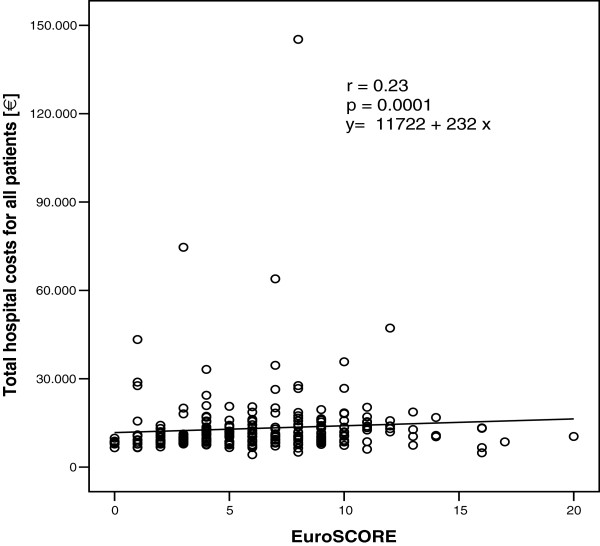
Linear regression for the EuroSCORE with hospital costs (n = 877).

**Figure 2:**
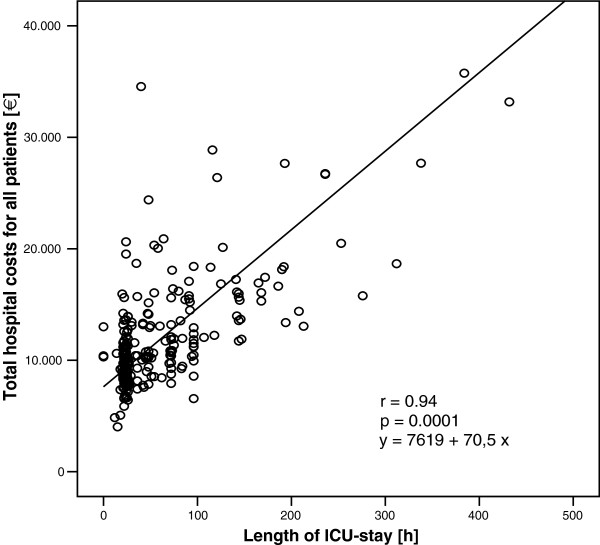
Linear regression for ICU length of stay with hospital costs (n = 877).

Recent studies with the EuroSCORE found a direct correlation between this risk model and hospital cost [5-7]. The *p*-values of the linear regression analysis were highly significant in all three studies (p < 0.0001). The correlation coefficients were r = 0.43 in the study of Sokolovic et al. [5] and r = 0.47 in both studies from Pinna Pintor et al. [6] and Nilsson et al. [7]. From a statistical point of view only coefficients r > 0.8 are an indicator of a good correlation [17]. A significant *p*-value means only, that the regression line is significantly different from a slope of zero. Figure 1 shows the Pearson’s correlation of EuroSCORE with total hospital cost, which demonstrates, that there is only a low correlation between the EuroSCORE and the hospital costs (r = 0.225). However, the regression line is significantly different from the zero value (*p* = 0.0001). To clarify this difference, we demonstrate in Figure 2 the Pearson’s correlation between the length of ICU stay and the total hospital cost. As expected, there is a good correlation between hospital costs and length of ICU stay (r = 0.94), with *p* < 0.01 as it is with EuroSCORE. The difference between both regression lines is obvious despite the same *p*-value in both cases.

Moreover, both Pinna Pintor et al. [6] and Nilsson et al. [7] used a log transformation of the dependent variable (hospital cost) in order to measure the relationship between EuroSCORE and hospital costs. The advantage of this transformation is that the influence of outliers is reduced and that the regression analysis looks better. This procedure gives an inflated sense of dependence of the hospital costs on the predictor variable (EuroSCORE) [18].

To our knowledge the only study with a high correlation coefficient was published by Kurki et al. [4] in 2002. They reported the correlation between the CABDEAL scoring system and hospital cost to be almost linear with a coefficient of r = 0.85 and a *p*-value < 0.0001. Unfortunately, this study has several limitations: First, the study of Kurki et al. [4] has a retrospective design. Secondly, three variables (arrhythmia, body mass index and creatinine) of a total of seven variables in the CABDEAL score were not constantly documented in the 30 different hospitals involved. Also, the hospital costs were calculated from hospital charge data. This so called “top-down” method divides the total budget into aliquots, thus calculating an average cost per patient. This is a simple method but has the disadvantage of being inaccurate at individual patient level. In our study we used the “bottom-up” approach, which is more time consuming but more reliable, as resource costs are added up from individual items of care [19]. Accordingly, we found the CABDEAL score to be associated with the worst correlation (see Tables 5 and 6) of all scores investigated.

**Table 5:** Correlation of scoring models with total hospital cost

**Score**	**r (Pearson)**	**p (Pearson)**	**r (Spearman)**	**p (Spearman)**
**Additive EuroSCORE**	0.225	< 0.01	0.254	< 0.01
**Cabdeal**	0.122	< 0.01	0.122	< 0.01
**French**	0.250	< 0.01	0.237	< 0.01
**Parsonnet**	0.149	< 0.01	0.296	< 0.01
**Ontario**	0.256	< 0.01	0.372	< 0.01
**Pons**	0.264	< 0.01	0.345	< 0.01

**Table 6:** Correlation of scoring models with reimbursement

**Score**	**r (Pearson)**	**p (Pearson)**	**r (Spearman)**	**p (Spearman)**
**Additive EuroSCORE**	0.133	< 0.01	0.111	< 0.01
**Cabdeal**	0.075	0.05	0.023	0.05
**French**	0.152	< 0.01	0.13	< 0.01
**Parsonnet**	0.102	< 0.01	0.176	< 0.01
**Ontario**	0.172	< 0.01	0.256	< 0.01
**Pons**	0.185	< 0.01	0.228	< 0.01

Ferraris et al. [20] found a poor correlation between the New York State risk model and hospital costs. A similar finding was reported by Riordan et al. [21], who demonstrated that the STS model is a poor predictor of costs on an individual patient level. The correlation coefficient in this study was r = 0.34. Both studies were confirmed by our data although we used different risk stratification models.

### Length of ICU-stay and total costs

All studies mentioned above [2-7,20,21] showed a good relationship between hospital costs and length of stay in the ICU, which was also confirmed by our study (r = 0.94, *p* = 0.0001, Figure 2). Time in the operating room (OR) is the most expensive part of hospitalization, however, OR time is relatively uniform between patients. The second most expensive stay is in the ICU which highly variable in this study (range was 14 – 1776 hours). Therefore, total hospital costs are likely to be correlated mainly with the ICU length of stay. The pre-operative hospitalization and non-ICU portion of the convalescence are much less expensive. However, this correlation between total cost and ICU length of stay can be calculated only after ICU discharge, which does not help the allocation of resources upon admission.

The cost of a non-fatal complication due to cardiac surgery is difficult to predict with existing models for risk-assessment. This may be because the models were designed to predict death rather than costs of morbid events. Also patients with complications do not necessarily have longer admission times, which introduce considerable variability when attempting to predict hospital cost from preoperative risk-assessment data. The failure of the six risk models tested in predicting costs is not surprising, since they were designed to predict mortality [10-13,22,23]. Thus, development of more accurate morbidity scores may be an option to achieve appropriate financial risk models.

## Conclusion

All six preoperative scoring models evaluated here fail to accurately predict the total costs of a cardiac surgery patient. Reconsidering the variable of these scores and their additive weight regarding the total hospital cost as an outcome is recommended to produce a reliable financial risk model.

### Consent

Written informed consent was not obtained from the patients for publication of this report or any accompanying images, since we report of a large population not about an individual patient. No image of an individual patient is accompanied.

## Abbreviations

EuroScore: European system for cardiac risk evaluation; ICU: Intensive care unit; OR: Operative room.

## Competing interests

The authors declare that they have no financial or non-financial competing interests.

## Authors’ contributions

AB: Conception and design; acquisition, analysis and interpretation of data; drafting the manuscript. FD: substantial contributions to conception and design; revising the manuscript critically for important intellectual content. AK: Data collection and final approval of the version to be published. TW: final approval of the version to be published. KH: substantial contributions to conception and design; interpretation of data; critically revising the manuscript for important intellectual content. All authors read and approved the final manuscript.
